# The Rarest of Rare Thymic Lesions: A 10-Year Surgical Pathology Experience

**DOI:** 10.3390/cancers13164056

**Published:** 2021-08-12

**Authors:** Fiorella Calabrese, Francesco Fortarezza, Federica Pezzuto, Francesca Lunardi, Giovanni Comacchio, Marta Sbaraglia, Giulia Pasello, Giuseppe Marulli, Angelo Paolo Dei Tos, Federico Rea

**Affiliations:** 1 Department of Cardiac, Thoracic, Vascular Sciences and Public Health, University of Padova, 35121 Padova, Italy; francesco.fortarezza@unipd.it (F.F.); federica.pezzuto@unipd.it (F.P.); francesca.lunardi@unipd.it (F.L.); giovannimaria.comacchio@aopd.veneto.it (G.C.); federico.rea@unipd.it (F.R.); 2 Department of Medicine, University of Padova, 35121 Padova, Italy; marta.sbaraglia@unipd.it (M.S.); angelo.deitos@unipd.it (A.P.D.T.); 3 Department of Surgery, Oncology, and Gastroenterology, University of Padova, 35121 Padova, Italy; giulia.pasello@unipd.it; 4 Department of Emergency and Organ Transplantation, University of Bari “Aldo Moro”, 70124 Bari, Italy; giuseppe.marulli@uniba.it

**Keywords:** thymus, surgical pathology, rare tumors

## Abstract

**Simple Summary:**

The thymus is the last organ in the human body to have its mechanisms fully understood. Its intrinsic complex morpho-functional structure reflects the complexity and variety of the pathological lesions of the gland. These include some variants of neoplastic and nonneoplastic processes, which are considered the rarest of the rare ones. The aim of this work is to describe the rarest lesions of the thymus in a large case series in order to update the epidemiology, to improve diagnostic awareness, and finally to promote a collaborative network between referral centers.

**Abstract:**

The thymus is a specialized primary lymphoid organ located in the midline pre-vascular mediastinum. The organ is the site of various pathological processes, neoplastic and not, whose rarity has not allowed in-depth studies on clinical or histological features of rarest and unusual variants. Herein, we report a 10-year Padova experience in the surgical pathology of the thymus, focusing on the pathological description of nonneoplastic lesions and rare epithelial and mesenchymal tumors recorded in our database, which comprises over 600 thymectomies. The extrapolated rare cases have been categorized into four groups that included 15 cysts, 18 carcinomas, 5 neuroendocrine tumors, and 2 soft tissue tumors. The cases are described from a clinical and pathological point of view and discussed in dedicated sections with a review of the most important literature. In this case, review series, we aim to update the epidemiology of these rare entities, improve diagnostic awareness, and finally, promote a collaborative network between referral centers.

## 1. Introduction

The thymus is a specialized primary lymphoid organ located in the midline anterosuperior mediastinum, essential for the maturation of T lymphocytes, which orchestrate adaptive immune responses [1]. The peculiar micro-architecture of the organ, based on the interconnection of epithelial and lymphoid cells (so-called thymocytes) with zonal compartmentation in the medulla and cortex, guarantees the fulfillment of this delicate function. The complex morpho-functional structure of the organ reflects the complexity and variety of the pathological lesions of the thymus. Indeed, the surgical pathology of the thymus encompasses a heterogeneous group of rare entities [2] which almost exclusively arise in the prevascular mediastinum with the exception of ectopic thymic tissue lesions. The most frequent thymus tumors in the adult population are represented by epithelial tumors, the thymomas [3], with an incidence of about 2 cases per 1,000,000 per year [4,5]. Since the first description of the association between myasthenia gravis (MG) and thymomas in the early 1900s [6], the study of thymic pathology has generated great scientific interest by several groups both in understanding the development of these rare neoplasms and especially in improving lesion classification. Indeed, few topics in pathology have been debated as much as the classification of thymic epithelial tumors, on which agreement has only been reached in recent years [7]. The study of thymic neoplasms is hampered by the rarity of the disease with a consequent negative impact on epidemiological and molecular studies. The field is further complicated by the presence of numerous and rare histological variants of epithelial, mesenchymal, lymphohematopoietic, and germinal origin with challenging pathological diagnoses, especially in limited small biopsy material. Recently, the development of national and international registers by leading international research groups (e.g., Thymic and Mediastinal Pathology Working Group of the European Society of Pathology) has allowed the collection of numerous data useful for conducting systematic studies. Moreover, international societies, such as ITMIG (International Thymic Malignancy Interest Group) have launched a prospective database (www.itmig.org, accessed date: 1 July 2021), and EURACAN (European Reference Network on Rare Adult Cancers) has provided infrastructure with a high level of multidisciplinary expertise for the diagnosis, management, and follow-up of patients with rare cancers, including thymic neoplasms. In 2014, a network named “TYME” (TYymic MalignanciEs) was founded in Italy with the aim of improving care and research in thymic tumors. Since 2021, this network has been enriched with a scientific regulation document, a steering committee, and two working groups for research and registry, all of them including referral members of the thoracic oncology team placed in Padova.

The improved knowledge of thymic pathology has been followed by the development of new surgical procedures. Historically, the gold-standard technique for thymectomy has been the transsternal approach, which ensures optimal exposure and availability of the complete dissection of the thymus and mediastinal fatty tissue [8]. Less-invasive surgical approaches have been developed over time, ranging from transcervical thymectomy or video-assisted thoracic surgery (VATS) thymectomy to the most modern techniques based on robotic-assisted thoracic surgery (RATS) that show a comparable perioperative outcome to open surgery in selected patients [9]. The execution of less-invasive surgical approaches could lead to an increase in thymectomies with a consequent more frequent detection of lesions currently defined as rare. Here, we report a 10-year Padova experience in the surgical pathology of the thymus, focusing on the pathological description of rare epithelial and mesenchymal tumors and nonneoplastic lesions recorded in our database, which comprises over 600 thymectomies.

## 2. Materials and Methods

All thymectomy cases in our center over the last decade (January 2011–December 2020) were reviewed, and the specimens were updated according to the recent World Health Organization [2] classification description and to both the Masaoka–Koga and TNM (eighth edition) staging systems. Cases with the final diagnosis of “thymic residues”, “thymic hyperplasia”, “thymic atrophy”, and “thymoma” were excluded. Four types of rare thymic lesions were detected: (1) thymic cysts, (2) thymic carcinomas (TC), (3) thymic neuroendocrine tumors (TNET), and (4) thymic soft tissue tumors. These lesions are described below in distinct sections, including a review of the literature, based on the Pubmed Medline database using the keywords of specific lesions and reporting all anecdotic descriptions.

## 3. Results

**Figure 1:**
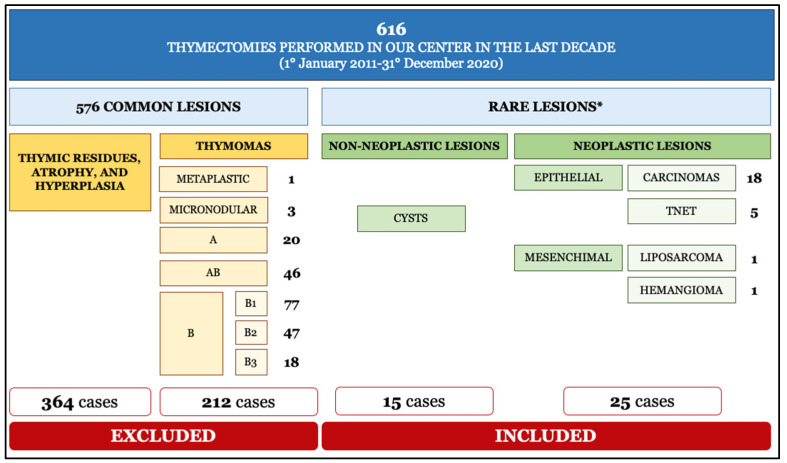
Histopathological classification of thymectomies performed at the Padova University Hospital in the last decade. * Lesions of concern in the present study. Abbreviations. TNET: thymic neuroendocrine tumors.

A total of 616 thymectomies were performed in our center over the last 10 years (Figure 1). After clinical and histopathological re-evaluation, 40 (6%) were classified as rare histotypes and variants, both neoplastic and nonneoplastic, and included 15 thymic cysts, 18 TC, 5 TNET, and 2 soft tissue tumors. The remaining cases (576, 94%), which included thymomas (212, 34%) and thymic residues, atrophy, or hyperplasia (364, 59%), were out of the scope of the study.

### 3.1. Thymic Cysts

**Figure 2:**
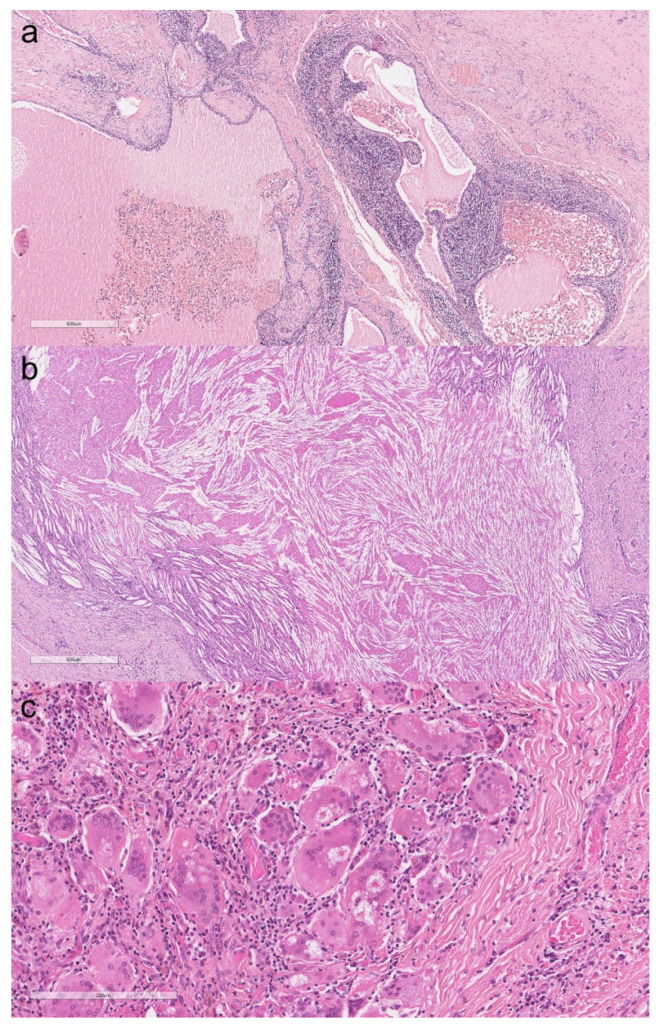
Multiloculated thymic cyst with thickened, fibrotic, and inflamed walls. The cystic spaces are filled with serum fluid and blood (**a**, hematoxylin and eosin, original magnification ×40). Thymic cyst with cholesterol granuloma (**b**, hematoxylin and eosin, original magnification ×40). Giant cell granuloma with several asteroid bodies around the wall of a thymic cyst (**c**, hematoxylin and eosin, original magnification ×200).

The main demographics and histopathological features of the patients with thymic cysts are listed in Table 1. Patients had a median age of 51 years and were slightly predominantly males (60%). Clinically, most cases were asymptomatic and incidentally detected. In four cases, the patients had MG, which clinically improved after the thymectomy. Macroscopically, the cysts were between 1 and 8 cm in size (median: 4 cm). Eight cysts were unilocular and seven multilocular. Cholesterol granulomas were evident in seven cases, most frequently in multilocular cysts. The cystic wall lining consisted predominantly of squamoid or cuboidal monolayer epithelial cells. In one case, there was mucinous metaplasia of the epithelium, and in another, the cuboidal lining appeared multilayered. The contiguous thymic parenchyma showed physiological adipose tissue involution in eight cases. In the remaining samples, features of epithelial or lymphatic-follicular hyperplasia were also evident, three of which were associated with MG. The subsequent follow-up was unremarkable for all patients. The most representative histological pictures of thymic cysts are grouped in Figure 2.

### 3.2. Thymic Carcinoma

**Figure 3:**
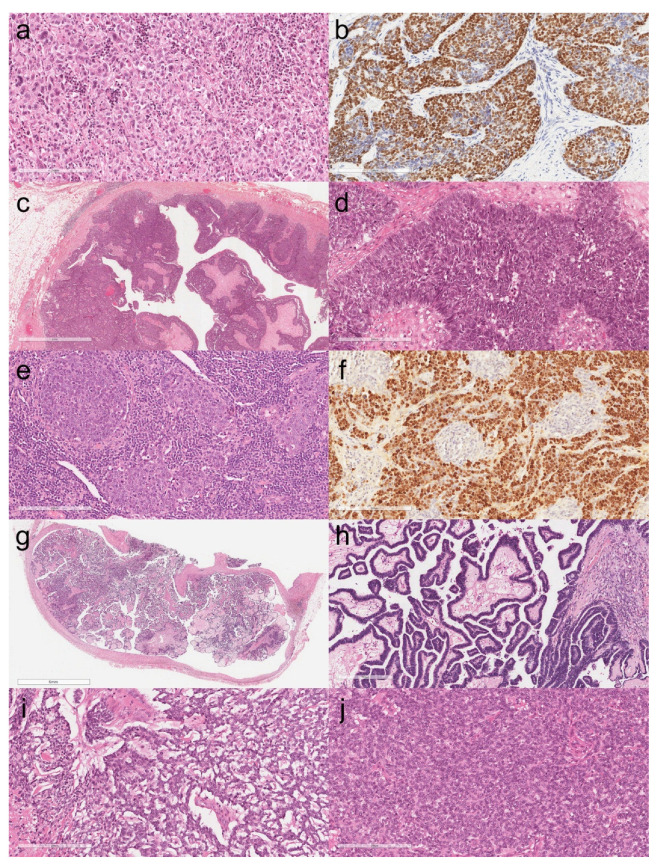
Non-keratinizing squamous cell carcinoma of the thymus (**a**, hematoxylin and eosin, original magnification ×200). The neoplastic nuclei were strongly positive for p40 (**b**, immunoperoxidase staining, original magnification ×200). Basaloid carcinoma in the context of multiloculated cyst (**c**, hematoxylin and eosin, original magnification ×20). The peripheral palisading is appreciable at higher magnification (**d**, hematoxylin and eosin, original magnification ×200). Lymphoepithelial carcinoma of the thymus. A dense lymphocytic infiltrate accompanies the nests of carcinoma cells (**e**, hematoxylin and eosin, original magnification ×200). EBER in situ hybridization positive in the neoplastic nuclei (**f**, original magnification ×200). Panoramic view of low-grade papillary adenocarcinoma (**g**, hematoxylin and eosin, original magnification ×10). The tumor was composed of papillary structures lined with mildly atypical cells (**h**, hematoxylin and eosin, original magnification ×100). Mucoepidermoid carcinoma of the thymus (**i**, hematoxylin and eosin, original magnification ×200). High-grade area of the tumor showing solid growth with focal mucus-producing cells (**j**, hematoxylin and eosin, original magnification ×200).

The main clinical and histopathological features of patients with TC are listed in Table 2. The median age of patients was 62.5 years with a clear male predominance (15/18, 83%). The most frequent histotype was squamous carcinoma (11/18, 60%), with one case combined with B3 thymoma. Other rare histotypes include basaloid carcinoma associated with multilocular cysts (1/18, 6%), lymphoepithelial carcinoma (2/18, 11%), mucoepidermoid carcinoma (1/18, 6%), low-grade papillary adenocarcinoma (1/18, 6%), thymic carcinoma, and histotypes not otherwise specified (2/18, 11%). In all cases, cytokeratin and at least two immunohistochemical markers supporting the diagnosis were positive in the neoplastic cells. The p63/p40 markers were positive in almost all cases (16/18, 88%), with the exception of low-grade papillary adenocarcinoma and one thymic carcinoma with aberrant expression of TTF1 that had been previously published by our group [10]. The two cases of lymphoepithelial carcinoma showed nuclear positivity for Epstein–Barr encoding region (EBER) in situ hybridization. Seven cases had advanced status at diagnosis with diffuse lymph node and/or distant metastases (Masaoka–Koga stage IVb). In three of these cases, patients died due to the disease. In nine cases (9/18, 50%), recurrence of the disease was reported. Representative histological images of TCs are grouped in Figure 3.

### 3.3. Thymic Neuroendocrine Tumors

**Figure 4:**
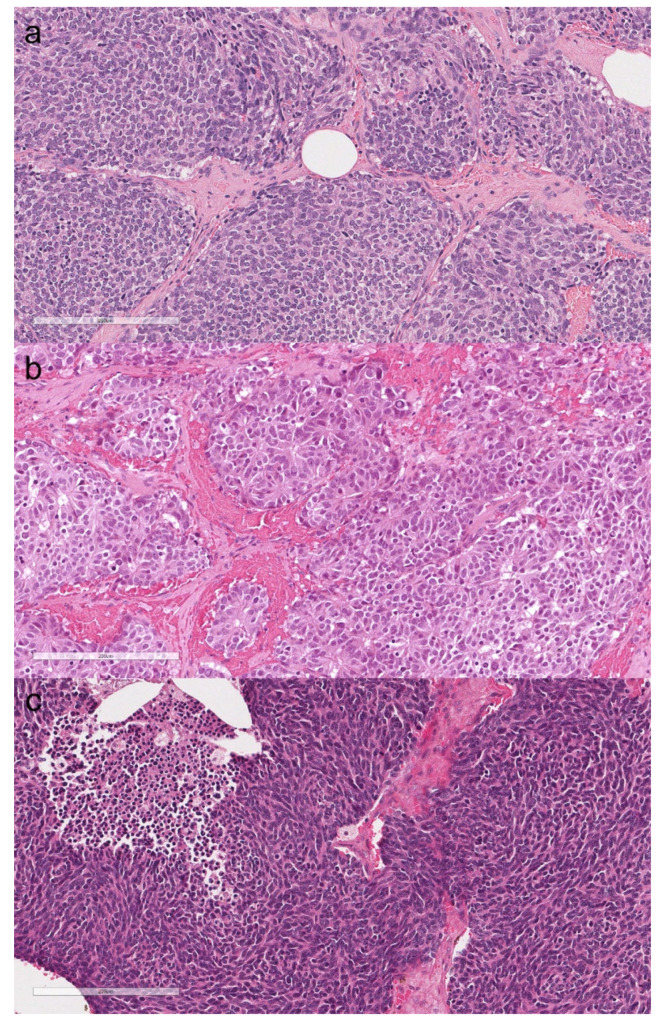
Typical carcinoid. Organoid proliferation of monomorphic neuroendocrine cells without mitotic activity nor necrosis (**a**, hematoxylin and eosin, original magnification ×200). Atypical carcinoid. Rosettoid proliferation of neuroendocrine cells showing mild nuclear pleomorphism. Two mitoses are present in the field (**b**, hematoxylin and eosin, original magnification ×200). Small cell carcinoma. Proliferation of small cells with necrosis and brisk mitotic activity (**c**, hematoxylin and eosin, original magnification ×200).

The main clinical and histopathological features of the patients with TNET are listed in Table 3. The median age of patients was 46 years with a clear female predominance (4/5, 80%). All the tumors expressed at least one neuroendocrine marker (e.g., Chromogranin A and Synaptophysin), and CD117 immunostaining was positive in four cases. The tumors were classified as low-grade (typical carcinoid), intermediate-grade (atypical carcinoid), and high-grade (small cell carcinoma) according to the latest international guidelines (mitotic count, assessment of necrosis, and morphological features) [2]. The patients with atypical carcinoid and small cell carcinoma died due to the disease. The most representative histological images of thymic NET are grouped in Figure 4.

### 3.4. Thymic Soft Tissue Neoplasms

#### 3.4.1. Cavernous Hemangioma of the Thymus

**Figure 5:**
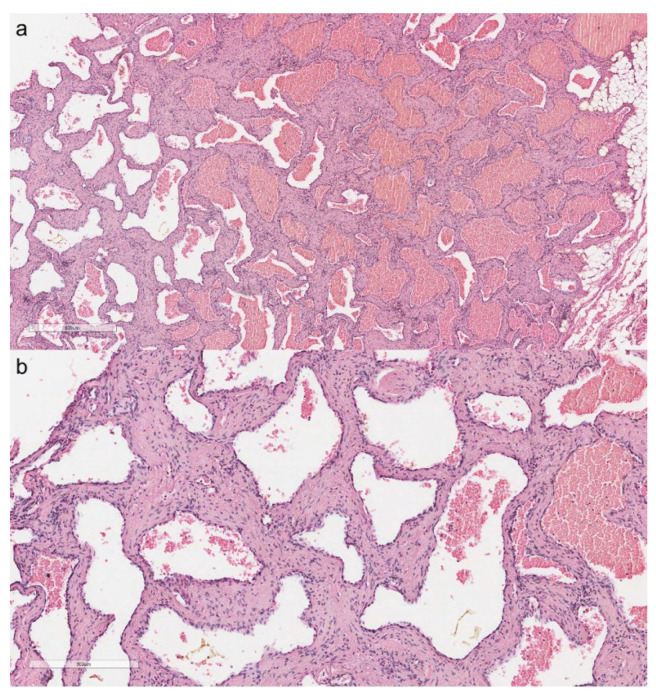
Cavernous hemangioma (**a**, hematoxylin and eosin, original magnification ×40). Dilated vessels lined by a single layer of flat endothelial cells were evident. Vascular spaces were separated by fibrous septa containing small vessels. No cytologic atypia or mitoses were detected (**b**, hematoxylin and eosin, original magnification ×100).

A 69-year-old female presented with ocular myasthenia, and otherwise, her medical history was unremarkable. The chest computed tomography (CT) scan showed a rounded mass of 1.5 cm in the prevascular mediastinum, compatible with thymoma. The patient underwent robotic thymectomy. Grossly, the nodule was well-circumscribed with a reddish-brown, spongy/honeycombed cut surface. Histology revealed dilated and thin-walled vessels lined by a single layer of flat endothelial cells. Vascular spaces were separated by fibrous septa containing small vessels (Figure 5). No cytologic atypia or mitoses were detected. The contiguous thymus showed fatty involution. The diagnosis of cavernous hemangioma of the thymus was made. The subsequent follow-up was unremarkable.

#### 3.4.2. Dedifferentiated Thymoliposarcoma

**Figure 6:**
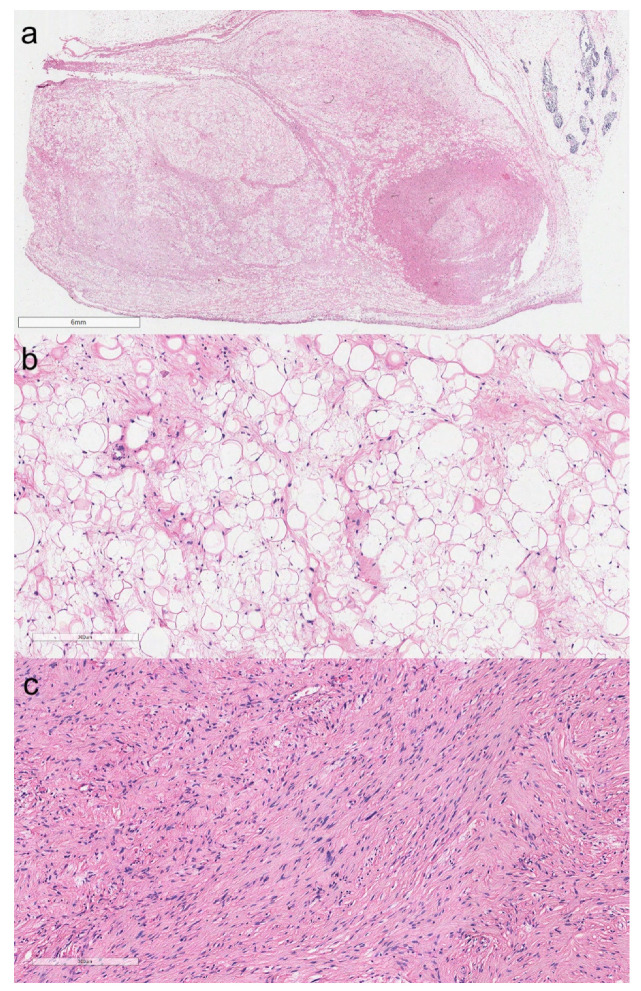
Dedifferentiated thymoliposarcoma. At the top right, thymic residues are visible (**a**, hematoxylin and eosin, original magnification ×10). The neoplasm was composed of a well-differentiated liposarcoma (**b**, hematoxylin and eosin, original magnification ×200), showing abrupt transition to a non-lipogenic spindle cell sarcoma (**c**, hematoxylin and eosin, original magnification ×200).

A 40-year-old man underwent radiological examinations for a persistent cough. Chest CT showed two masses of the right upper lobe and prevascular mediastinum. Transbronchial biopsy showed a poorly differentiated spindle cell neoplasm with immunohistochemical expression of CD56 and a high proliferative index (Ki67: 80%). The differential diagnoses included a neuroendocrine and mesenchymal neoplasm. The patient underwent lobectomy and thymectomy. The thymus was almost entirely replaced by a lipomatous neoplasm with nodular areas of increased consistency (Figure 6). The lung tumor shared the same features as those of the thymic nodules. Histology showed the thymic tumor consisted of a well-differentiated liposarcoma component with abrupt transition to a high-grade, non-lipogenic spindle cells sarcoma. The lung tumor consisted entirely of the latter component. Diffuse nuclear expression of Murine Double Minute 2 -MDM2 was detected in the neoplastic cells. The final diagnosis of dedifferentiated liposarcoma of the thymus was made. The patient was free of disease at the last follow-up (13 months).

## 4. Discussion

### 4.1. Thymic Cysts

Thymic cysts were first described in 1850 [11] and represent a very rare lesion accounting for about 1% of all mediastinal cysts [12]. The dimensions are very variable, ranging from 1 to 18 cm [13,14]. They are either acquired or congenital and, although they mainly affect the prevascular mediastinum, they may be localized along the normal descent route from the base of the neck to the diaphragm during thymus embryogenesis. Typically, congenital cysts are unilocular with a thin wall and derive from embryonal thymic tissue [15]. They often contain serous fluid and are lined by a cuboidal, flat, columnar, or stratified squamous epithelium. In very rare cases, foci of mucinous metaplasia of the epithelium may be present, as in case 6. To the best of our knowledge, there is only one case in the English literature reporting this unusual morphological finding as a potential cause of diagnostic pitfall [16]. Multiloculated thymic cysts are characterized by thick and fibrous septa, considered reactive in nature [13] and, consequently, may be associated with other pathological processes, such as tumors, infections, or radiotherapy [17]. Marked inflammation, cholesterol granulomas, and granulation tissue often coexist. Thymic cysts, especially the congenital subtypes, are typical of the pediatric age, although they may be diagnosed at an older age since they are often asymptomatic [18]. Indeed, as also in our series (9/15 asymptomatic), thymic cysts are frequently diagnosed as incidentalomas during radiological examinations performed for other reasons [19] or during surgery [20]. Some symptoms, such as cough, dyspnea, and chest pain, may be due to the size of the cysts, which causes pressure and/or a mass effect, as has been observed in two cases. MG represents a very rare complication of thymic cysts. In the literature, seven cases with MG have been reported, either unilocular or multilocular cysts [21,22,23,24,25], but little is known about the pathogenesis and progression of MG after surgery. In three cases with MG from our series, the contiguous thymic parenchyma to the cysts showed follicular or epithelial hyperplasia. Such features are well known to be related to MG and could explain the symptoms in these patients. In the other cases previously published, there is no specific description of the contiguous thymic parenchyma. The clinical improvement of all patients with MG confirms the usefulness of thymectomy in non-thymomatous MG patients [26].

### 4.2. Thymic Carcinoma

TCs are rare neoplasms with an incidence of 0.07–0.38/100,000/year [27]. They mainly occur in the prevascular mediastinum comprising 7.5% of the lesions at this site [3], and mainly affect males in the fifth–sixth decade [28,29]. The neoplastic mass can cause symptoms (such as dyspnea, chest pain, or superior vena cava syndrome) related to its growth and the compression of the surrounding anatomical structures. Compared to thymomas, an association with MG and other paraneoplastic syndromes has rarely been reported (<2%) [30]. As for thymomas, surgery is the mainstay of treatment in case of potentially resectable disease. However, TCs are characterized by aggressive clinical behavior and present with an advanced stage at the time of diagnosis (46.5% in stage IV); therefore, a multimodal therapy is necessary [31]. Cisplatin–anthracycline or cisplatin–etoposide chemotherapic combinations are recommended as (neo)adjuvant treatment or in case of extensive disease. Recent studies with anti-PD-L1 immunotherapy showed promising results but still need confirmation [32]. Radiotherapy is considered in unresectable disease or as adjuvant treatment in stage II and stage III disease with R0/R1 resection [31]. Despite multimodal therapy, TCs show a high rate of recurrence (51%), and the overall survival rate at 10 years is approximately 40% in thymic carcinoma [33]. The most relevant prognostic factors for overall survival include complete resection, Masaoka–Koga stage, and TNM-based pathological staging [34]. Differently from thymoma, thymic carcinoma types seem to have no prognostic relevance [34]. The latest WHO histologic classification [2] classified thymic carcinoma based on morphological and, sometimes, immunohistochemical and molecular analyses. Generally, most of the histotypes such as squamous or mucoepidermoid are morphological (at histology and immunohistochemistry) to the counterpart of another organ. Therefore, searching for a clinical-radiological correlation and for immunomarkers of thymic origin is mandatory to exclude mediastinal metastases from cancers of other sites [35]. Squamous cell carcinoma (SCC) is the most common histotype of thymic carcinoma accounting for 70–80% of all TC, similar to what emerged from our case series [2,34,36]. The etiology is still largely unknown. Several cytogenetic and molecular alterations have been reported in the literature, including chromosome imbalances [37], mutations in epigenetic regulatory genes [38], and DNA methylation of several genes [39]. KIT mutation is present in about 10% of all thymic SCC and may represent a molecular target for tyrosine kinase inhibitor therapy, as found in several case reports [40,41] and cohort studies [42]. Histologically, SCC of the thymus resemble SCC of other sites: they may or may not be keratinized, although prominent keratinization is rarely present in neoplasms of thymic origin [2]. Rarely, thymic carcinomas may be combined with other thymic epithelial neoplasms, especially with thymomas B2/B3 [43]. One case of SCC combined with a B3 thymoma emerged from our series, documenting this unusual heterogeneous entity. A rare variant (<5% of all carcinomas) of SCC of the thymus is the basaloid carcinoma, of which about 40 cases have been described in the literature [2]. Basaloid carcinoma is characterized by small- and medium-sized cells organized in solid, cystic, or papillary patterns often combined with a peripheral palisade. In half of the cases reported in the literature, multilocular cysts are also described, similar to our case. It is thought that the cysts may represent a precursor lesion [44]. We have described two cases of thymic lymphoepithelial carcinoma in two males, one 34 years old and one 73 years old. This tumor accounts for 1–6% of all thymic carcinomas [36,45,46]. It can affect individuals of all ages but has a bimodal peak incidence at 14 and 48 years. The key diagnostic histological feature is the presence of a dense lymphocyte infiltrate accompanying nests of neoplastic cells resembling undifferentiated carcinoma of the nasopharynx. An association with EBV infection has been described in about half of the cases [47], mainly in young adults. The search for in situ hybridization for EBER is a useful diagnostic support to corroborate the diagnosis of this rare histotype, as shown by our two positive cases. It is a very aggressive neoplasm, even if a complete resection and a low pathological stage may have a favorable prognosis, as indicated by case 5. Immunohistochemistry is a useful and mandatory tool for the final assessment of thymic origin. TC cells, as well as their nonneoplastic counterpart represented by thymic cortical and medullary epithelial cells, are typically positive for cytokeratin, PAX8, and p63/p40. These markers are useful to differentially diagnose other carcinomas, including neuroendocrine tumors that may share several morphological features with thymic carcinomas or with lymphoproliferative processes, preferably using the p40 marker rather than p63, which may be positive in mediastinal large B-cell lymphoma [48]. Other markers, such as CD5, CD117, and GLUT1, favor the diagnosis of thymic carcinoma rather than thymoma if they are expressed by neoplastic epithelial cells [49]. Most of the cases we describe comply with these rules, with the exception of extremely rare tumors and variants. Low-grade papillary adenocarcinoma is a primitive tumor of the thymus characterized by papillary and tubulopapillary fronds composed of slightly atypical cells, completely or partially encapsulated with cystic and/or solid areas. It could be associated with A or AB thymoma. Immunostaining is not useful in assessing the thymic origin of the neoplasm because LGPA is negative for p40, p63, and PAX8 and is variably and focally positive for CD117 and CD5. The latter is not indicative of a thymic origin in adenocarcinomas [50]. Therefore, to determine the thymic primitivity of these neoplasms, it is necessary to exclude metastases from other sites, such as the thyroid, lung, or gastrointestinal tract. The thymus may rarely be the site of salivary gland tumors. The most common histotype is represented by mucoepidermoid carcinoma morphologically and is immunophenotypically analogous to the salivary counterpart, and is characterized by three different cell populations: mucus-secreting, intermediate, and squamous cells. It accounts for about 2.5% of all thymic neoplasms [34]. An association with multilocular cysts [51] and thymomas [52] has been reported. The presence of *CRTC1–MAML2* gene fusion may be found in thymic mucoepidermoid carcinoma, although less frequently than in salivary gland mucoepidermoid carcinomas [53]. When it is impossible to classify neoplasms in one of the thymic carcinoma entities, the WHO classification suggests diagnosing them as thymic carcinomas, not otherwise specified (TC, NOS) [2]. In our case series, we described two cases of TC, NOS, one of which showed an aberrant expression of TTF-1, which led to the erroneous diagnosis of lung adenocarcinoma on the biopsy sample. This case is an example of how challenging and tricky the histopathological diagnosis of thymic neoplasms can be, which should thus include a wide correlation of clinical–radiological, histological, and immunohistochemical data to achieve a correct diagnosis [10].

### 4.3. Thymic Neuroendocrine Tumors

Primary neuroendocrine tumors of the thymus are rare, accounting for less than 5% of all mediastinal masses [54] and for 0.4% of all neuroendocrine tumors [55]. Up to 25% of thymic neuroendocrine tumors arise in patients with multiple endocrine neoplasia type 1 (MEN-1). Whenever feasible, radical surgical resection is indicated. However, the recurrence rate is also high in R0 resections, and the prognosis is poor, as low-grade thymic neuroendocrine tumors present 5-year and 10-year survival rates of 50% and 9%, respectively, whereas no high-grade survives at 5 years. In the case of advanced tumors, aggressive multidisciplinary treatment is indicated. Platinum-based treatments combined with surgical resection, when indicated, are the standard for metastatic tumors, although these tumors show a rather poor response rate to the available chemotherapy regimens, and the response to radiation alone is poor [56].

Neuroendocrine neoplasms of the thymus are classified in the same way as neuroendocrine neoplasms of the lung, recognizing four groups with different prognostic significance based on mitotic count, necrosis, and morphological findings: typical carcinoid (low-grade; <2 mitoses/2 mm^2^, no necrosis), atypical carcinoid (intermediate-grade; necrosis and/or 2–10 mitoses/2 mm^2^), large cell neuroendocrine carcinoma (high-grade, no small cell cytology, neuroendocrine markers, necrosis, >10 mitoses/2 mm^2^), and small cell carcinoma (high-grade; small cell cytology, >10 mitoses/2 mm^2^) [2]. The choice of a similar classification for lung and thymus neuroendocrine neoplasms is due to the numerous aspects shared by these tumors: morphological features, absence of correlation with smoking for typical and atypical carcinoids, similar prognosis among the various groups, and development of carcinoids both in the lungs and in the thymus of patients with multiple neuroendocrine neoplasia type 1 [57,58]. However, some differences need to be pointed out. Thymic carcinoids affect males more frequently than lung carcinoids, which occur more frequently in females. Moreover, high-grade neuroendocrine neoplasms of the thymus show a weaker link with smoking than their pulmonary counterparts. The reasons for these differences are still unknown, even if molecular pathology studies have shown some differences between neuroendocrine neoplasms of the thymus and lung, especially in the typical and atypical carcinoid groups [55,59]. Although there are little data in the literature, some evidence suggests that thymic neuroendocrine tumors are characterized by a more aggressive biological behavior compared to neuroendocrine neoplasms of other sites, such as the lung or gastrointestinal tract [60]. Similar to other thymic epithelial malignancies, the pathological stage and complete resection of the tumors are the strongest prognostic factors [60]. A debated topic in the literature is the role of histology on the overall survival time of these neoplasms. Several case series have pointed out a possible prognostic role of histologic classification [61,62,63], with the exception of one study by Filosso et al. based on a large number of cases (205) [64] that excluded a prognostic role of histology on both overall survival and cumulative incidence of recurrence. In our series, three patients died of disease, two of those with atypical carcinoid. Interestingly, case 1 showed a poor prognosis despite the very early stage of the disease (pT1aN0M0), highlighting the intrinsic aggressiveness that these neoplasms may have.

### 4.4. Soft Tissue Thymic Tumors

The mediastinum can be the site of virtually any soft tissue tumor. However, the preferential thymic localization is very rare and is a characteristic of only a selected category of histotypes. The most frequent mesenchymal tumor of the thymus is thymolipoma, an encapsulated tumor composed of mature adipose tissue with interspersed nonneoplastic thymic tissue. It accounts for 2–9% of all thymic neoplasms and may occur at any age without sex predominance [65,66]. Its biological nature is unknown. In one case, a translocation involving the High Mobility Group AT-Hook 2-*HMGA2* gene on chromosome 12q15 was detected, supporting the theory that thymolipoma is a neoplasm of thymic fat [67]. The malignant counterparts of lipoma, the liposarcoma, and its variants rarely develop in the mediastinum. Although in most cases they are metastases or direct extensions from retroperitoneal tumors [68], liposarcomas are the most common primary sarcomas of the prevascular mediastinum [69]. In rare cases, direct origin from the thymus has been demonstrated (i.e., thymoliposarcoma) [70,71], similar to the case described here. The prognosis of liposarcoma is poor and correlates with tumor type; its mortality rate ranges from 30% to 50% [70]. The other thymic mesenchymal lesion that emerged from our series was a cavernous hemangioma. It is now considered to be a venous malformation rather than a true neoplastic process [72]. In the literature, 10 cases of thymic hemangioma have been reported [73] in which no sex predominance nor preferential age emerged. Other mesenchymal neoplasms of thymic origin are extremely rare and are described in the literature as case reports. These include solitary fibrous tumor [74], lymphangioma [75,76], angiosarcoma [77], gangliocytic paraganglioma [78], and neuroblastic tumors associated with a thymic cyst [79] or with the syndrome of inappropriate antidiuretic hormone secretion (SIADH) [80,81,82].

## 5. Conclusions

The pathology of the thymus is a fascinating field of research. The rarity of the thymic lesions did not allow us to thoroughly study the epidemiological aspects, clinical characteristics, or biological behavior of some of these rare neoplasms, such as those described in this paper. Reporting a detailed description of these cases is extremely important to update the epidemiology of these rare lesions, to improve diagnostic awareness, and above all, to promote a collaborative network between referral centers.

## Figures and Tables

**Table 1:** Main clinical and histological features of 15 patients with thymic cysts.

Case	Age	Sex	Clinic	Size (cm)	Cyst Histology	Thymus Histology
1	65	M	Asymptomatic	5	Unilocular	Fatty involution
2	39	M	MG	4	Multilocular with CG	Follicular hyperplasia
3	61	M	Asymptomatic	3.5	Unilocular with CG	Fatty involution
4	70	M	Asymptomatic	8	Multilocular with CG and calcification	Fatty involution
5	41	F	MG	6	Multilocular	Follicular hyperplasia
6	40	M	Asymptomatic	6	Unilocular with epithelial mucinous metaplasia	Fatty involution
7	57	F	Asymptomatic	8	Multilocular with granulation tissue	Epithelial hyperplasia
8	44	F	Dyspnea	6.5	Unilocular with CG	Follicular hyperplasia
9	49	F	Asymptomatic	4	Multilocular with CG	Fatty involution
10	70	M	MG	3	Unilocular	Epithelial hyperplasia
11	51	M	Asymptomatic	1	Unilocular with stratified cuboidal epithelium	Fatty involution
12	69	M	MG	1.4	Multilocular with CG	Fatty involution
13	26	F	Chest pain	3.7	Multilocular with CG	Epithelial hyperplasia
14	62	M	Asymptomatic	2	Unilocular	Fatty involution
15	30	F	Asymptomatic	5.1	Unilocular with CG	Epithelial hyperplasia

Abbreviations. CG: cholesterol granuloma; F: female; M: male; MG: myasthenia gravis.

**Table 2:** Main clinical and histological features of 18 patients with thymic carcinoma.

Case	Age	Sex	Histotype	Immunohistochemistry	M–K	TNM (Stage)	Recurrence	Follow-Up †	Status
1	52	F	SCC	CK+; CD5+−; CD117+; p63+	IVb	T3N0M1a (IVA)	Yes	60	DOD
2	62	M	BC	CK+; CD5+−; CD117+; CGA−; Syn−; p63+	IIb	T1aN0 (I)	Yes	113	AWD
3	65	M	SCC	CK+; CD5+; CD117+; p63+	IVb	T1bN1M1b (IVB)	Yes	22	DOD
4	60	F	SCC	CK+; CD5+; CD117+; p63+	IVb	T3N2M0 (IVA)	Yes	18	DOD
5	73	M	LEC	CK+; CD5+; CD117+; p63+; Syn+−; EBER+	IIb	T1aN0Mx (I)	No	77	NED
6	63	M	SCC	CK+; CD5+; CD117+; p63+	III	T2N0M0 (II)	Yes	75	AWD
7	84	M	SCC	CK+; CD5+−; CD117+−; p63+; GLUT1+	III	T3N0M0 (IIIA)	No	66	NED
8	73	M	SCC	CK+; CD5+−; CD117+; p63+; GLUT1+	IIb	T1aN0M0 (I)	No	16	DOC
9	75	M	c-SCC ‡	CK+−; CD5+−; CD117+; p63+; GLUT1+−	IVb	T3N1M1a (IVA)	No	63	NED
10	72	M	SCC	CK+; CD5+−; CD117+; p63+; GLUT1+	III	T3N0M0 (III)	No	61	NED
11	67	M	SCC	CK+; CD5+; CD117+; p63+; p40+; GLUT1+	III	T3N0M0 (III)	Yes	29	AWD
12	62	M	SCC	CK+; CD5+−; CD117+; p40+; GLUT1+−	III	T3N0M0 (III)	No	23	NED
13	56	F	LGPA	CK+; CD5+−; CD117−; GLUT1+; TTF1−; CDX2−, p40−	IIa	T1aN0M0 (I)	No	21	NED
14	70	M	SCC	CK+; CD5−; CD117+; p63+; GLUT1+	III	T1bN0M0 (I)	No	18	NED
15	62	M	MEC	CK+; p63+−; CK7+−, CD117−; CD5−; GLUT1−	IVb	T3NxM1b (IVB)	Yes	23	AWD
16	34	M	LEC	CK+; CD5+; CD117+; p40+; GLUT1−; EBER+	IVb	T3N0M1b (IVB)	Yes	11	AWD
17	58	M	TC, NOS	CK+; CD5−; CD117+; p63+; GLUT1+	IIa	T1aN0M0 (I)	No	9	NED
18	49	M	TC, NOS	CK+; CD5+; CD117+; p63−; GLUT1+−; TTF1+	IVb	T3N2M0 (IVA)	Yes	17	AWD

† Last follow-up in months. ‡ Combined-SCC with B3 thymoma. Abbreviations. AWD: alive with disease; BC: basaloid carcinoma; CGA: chromogranin A; CK: cytokeratin; c-SCC: combined squamous cell carcinoma; DOC: died of other cause; DOD: died of disease; EBER: Epstein–Barr encoding region in situ hybridization; F: female; LEC: lymphoepithelial carcinoma; LGPA: low-grade papillary adenocarcinoma; M: male; MEC: mucoepidermoid carcinoma; M–K: Masaoka–Koga stage; NED: no evidence of disease; Syn: synaptophysin; TC, NOS: thymic carcinoma, not otherwise specified.

**Table 3:** Main clinical and histological features of five patients with TNET.

Case	Age	Sex	Histotype	Immunohistochemistry	M–K	TNM (Stage)	Recurrence	Follow-Up †	Status
1	53	M	AC	CK+, CGA+; Syn+; CD117+; Ki67: 10%	II	T1aN0M0 (I)	Yes	75	DOD
2	44	F	SCC	CK+, CGA−; Syn+; CD117+; Ki67: 70%	IVb	T3N1M1a (IVA)	No	0	DOD
3	47	F	AC	CK+, CGA−; Syn+; CD117+; Ki67: 20%	IVb	T3N1M1a (IVA)	Yes	51	DOD
4	46	F	TyC	CK+, CGA+; Syn+; CD117+; Ki67: 4%	IIa	T1aN0M0 (I)	No	51	NED
5	27	F	c-NET ‡	CK+, CGA+; Syn−; CD117−; Ki67: 1%	IVa	T3N0M1a (IVA)	Yes	28	AWD

† Last follow-up in months. ‡ Combined typical carcinoid with B1 thymoma. Abbreviations. AC: ctypical carcinoids; AWD: alive with disease; DOD: died of disease; c-NET: combined neuroendocrine tumor; CGA: chromogranin A; F: female; M: male; M–K: Masaoka–Koga stage; NED: no evidence of disease; SCC: small cell carcinoma; Syn: synaptophysin; TyC: typical carcinoid.

## Data Availability

No new data were created or analyzed in this study. Data sharing is not applicable to this article.
